# Supplementary material for: Splice-Junction-Based Mapping of Alternative Isoforms in the Human Proteome
Source: Cell Rep. Author manuscript; Available in PMC 2020 Jan 15. (PMC6961840; doi:10.1016/j.celrep.2019.11.026)
Supplement: 2 [file NIHMS1546469-supplement-2.zip › DF1/PXD010154/Thyroid/Thyroid_31_HIF1A_ALDQVLQFLR.pdf]

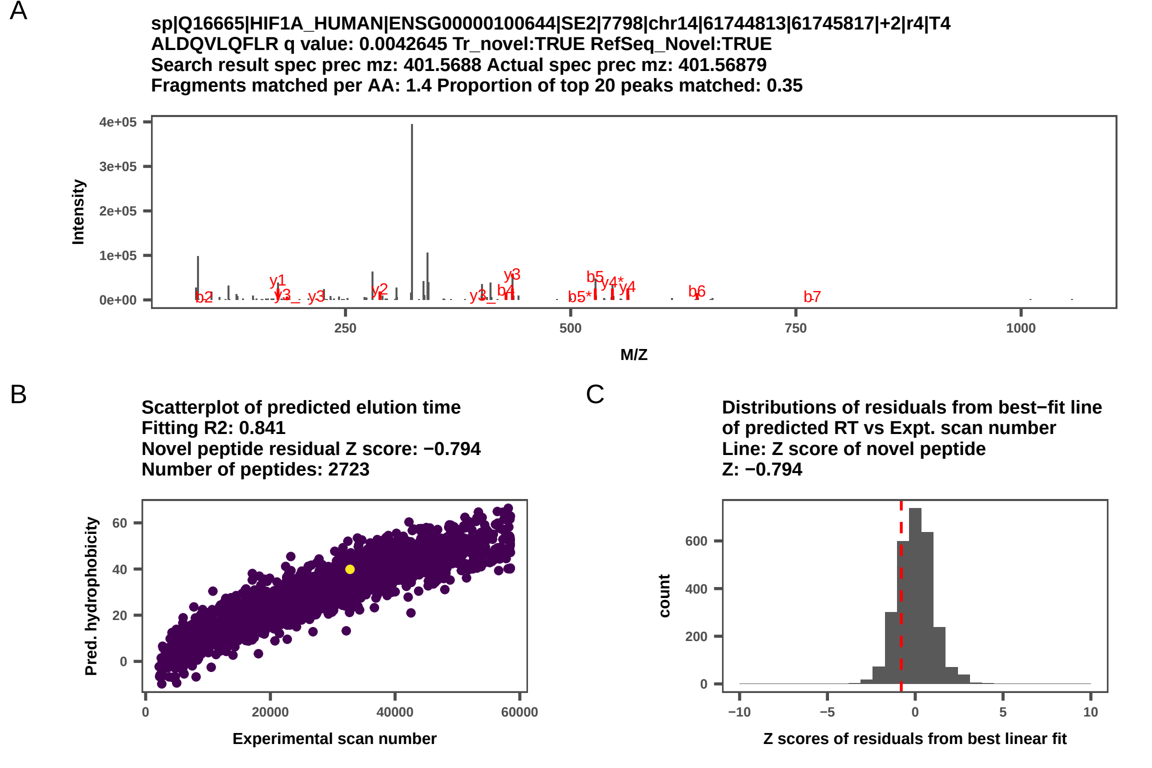
A
sp|Q16665|HIF1A_HUMAN|ENSG00000100644|SE2|7798|chr14|61744813|61745817|+2|r4|T4
ALDQVLQFLR q value: 0.0042645 Tr_novel:TRUE RefSeq_Novel:TRUE
Search result spec prec mz: 401.5688 Actual spec prec mz: 401.56879
Fragments matched per AA: 1.4 Proportion of top 20 peaks matched: 0.35
Intensity
4e+05
3e+05
2e+05
1e+05
0e+00
b2
y1
y3_
y3
y2
y3_
b4
y3
b5*
b5
y4*
y4
b6
b7
250
500
750
1000
M/Z
B
Scatterplot of predicted elution time
Fitting R2: 0.841
Novel peptide residual Z score: −0.794
Number of peptides: 2723
Pred. hydrophobicity
60
40
20
0
0
20000
40000
60000
Experimental scan number
C
Distributions of residuals from best−fit line
of predicted RT vs Expt. scan number
Line: Z score of novel peptide
Z: −0.794
count
600
400
200
0
−10
−5
0
5
10
Z scores of residuals from best linear fit
